# An international review of the patterns and determinants of health service utilisation by adult cancer survivors

**DOI:** 10.1186/1472-6963-12-316

**Published:** 2012-09-13

**Authors:** Charlene Treanor, Michael Donnelly

**Affiliations:** 1 Cancer Epidemiology & Health Services Research Group, Centre for Public Health, Queen’s University Belfast, Belfast, UK; 2 UKCRC Centre of Excellence for Public Health, Queen’s University Belfast, Belfast, UK

**Keywords:** Cancer survivor, Health service utilisation, Systematic review, Andersen Behavioural Model

## Abstract

**Background:**

There is a need to review factors related to health service utilisation by the increasing number of cancer survivors in order to inform care planning and the organisation and delivery of services.

**Methods:**

Studies were identified via systematic searches of Medline, PsycINFO, CINAHL, Social Science Citation Index and the SEER-MEDICARE library. Methodological quality was assessed using STROBE; and the Andersen Behavioural Model was used as a framework to structure, organise and analyse the results of the review.

**Results:**

Younger, white cancer survivors were most likely to receive follow-up screening, preventive care, visit their physician, utilise professional mental health services and least likely to be hospitalised. Utilisation rates of other health professionals such as physiotherapists were low. Only studies of health service use conducted in the USA investigated the role of type of health insurance and ethnicity. There appeared to be disparate service use among US samples in terms of ethnicity and socio-demographic status, regardless of type of health insurance provision s- this may be explained by underlying differences in health-seeking behaviours. Overall, use of follow-up care appeared to be lower than expected and barriers existed for particular groups of cancer survivors.

**Conclusions:**

Studies focussed on the use of a specific type of service rather than adopting a whole-system approach and future health services research should address this shortcoming. Overall, there is a need to improve access to care for all cancer survivors. Studies were predominantly US-based focussing mainly on breast or colorectal cancer. Thus, the generalisability of findings to other health-care systems and cancer sites is unclear. The Andersen Behavioural Model provided an appropriate framework for studying and understanding health service use among cancer survivors. The active involvement of physicians and use of personalised care plans are required in order to ensure that post-treatment needs and recommendations for care are met.

## Background

The number of cancer survivors (CSs) is increasing steadily due to several factors including improved medical treatment and an aging population [1]. However, there are relatively few studies about health service use by individuals with this chronic condition. The few studies that have been conducted in this field provide mixed results about the nature and extent to which CSs have poorer health and a greater need for services than primary care patients and other chronic disease groups [2,3]. There is a need to investigate health service utilisation in order to understand access to services, identify any service gaps and to improve organisational efficiency and cost-effectiveness [4]. The nature and type of services required by the CS population including effective and efficient ways in which to organise, deliver and facilitate services is relatively unexamined. The purpose of this paper is to present the results of a systematic review of the literature related to the patterns and determinants of the use of preventive health care services, hospital care and primary care by cancer survivors.

## Methods

**Figure 1:**
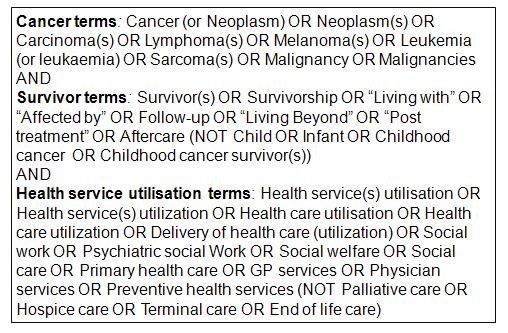
Full search strategy.

MEDLINE, CINAHL, PsycINFO, Social Science Citation Index and the SEER-MEDICARE online library of publications were searched from 1950 to August 2011. The search terms revolved around the key concepts: *cancer survivors* and *health service utilisation* (see Figure 1). The methodology adhered to guidelines by the Centre for Review and Dissemination [5]. Independent study selection was undertaken by two reviewers who achieved 98 % agreement. Data were extracted using a pre-specified pro-forma. 

Papers published in peer-review journals were included if participants were diagnosed with cancer in adulthood; had completed active treatment with curative intent and were not in receipt of palliative care. Non-melanoma skin CSs were excluded as both treatment and survival differ from other cancer sites [6]. The review included studies of key formal service components including primary care, hospital services, social services, mental health services (and their costs). Studies of eHealth systems, lay-led supportive services, dentistry and complementary and alternative medicine were excluded. Many CSs use these other types of services, particularly complementary and alternative therapies in order to manage the long-term morbidity associated with cancer. However, these service types were excluded from the review due to resource limitations and each service type would warrant a separate review.

The STrengthening the Reporting of OBservational studies in Epidemiology (STROBE) checklist was supplemented with a survey appraisal checklist in order to appraise the methodological quality of the full range of study types [7,8].

The Andersen Behavioural Model provided the theoretical and organisational framework for the review and synthesis of studies. Service use by CSs was explained in terms of three main components: characteristics which *predispose* (e.g. age, sex and health beliefs) an individual to use health care; *enabling* characteristics (e.g. resources) that facilitate access to health care; and *need* in the form of an illness or symptoms that require care (e.g. follow-up screening to detect cancer recurrence or metastases). This model was used as the organisational framework because it was developed as an explanatory framework explicitly for health service utilisation; it has been applied across various types of health services and health care systems and there is a large associated international literature on its performance. It encompasses individual characteristics and takes into consideration the context in which health care occurs.

## Results

### Study characteristics

**Figure 2:**
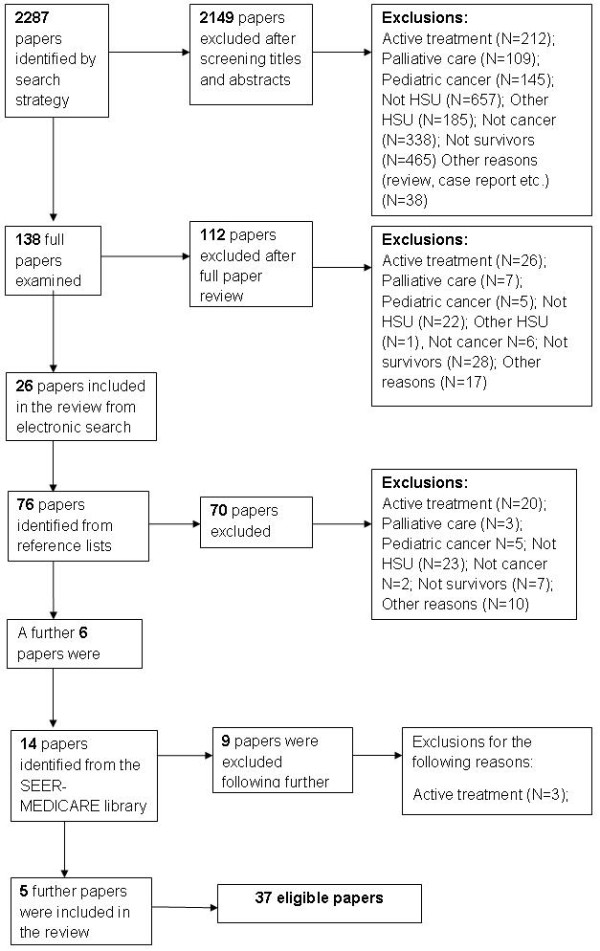
Search and screening results.

A total of 38 studies were included in the review (see Figure 2); studies took place in the USA (27), UK (3), Netherlands (3), Canada (3), France (1) and Denmark (1). Breast CSs were the population of interest in the majority of studies (18); other studies comprised colorectal CSs (11), uterine CSs (1) or survivors from a number of cancer sites (7). See Tables 1 and 2 for study characteristics. The country of study origin is stated in the results below unless studies were conducted in the USA. Studies focussed on primary care (26), post-active treatment cancer surveillance (15), preventive care, e.g. influenza vaccination, (8), mental health service use, e.g. psychologists, (3) and hospital care, including inpatient and outpatient services (2). Use of social services was not a primary focus of any of the identified papers. The studies investigated the prediction of service utilisation but not the nature and extent of service integration and coordination. Quality appraisal scores ranged from 11 to 20, (mean = 17), indicating that the majority of studies (n = 25) were very good quality. No study exclusions were made based on quality.

**Table 1:** Study Characteristics

**Author**	**Year**	**Country**	**Sample characteristics**	**Analysis**	**Variables/Measures**	**Outcome**	**Quality appraisal**
Andersen and Urban [36]	1998	USA	Breast cancer survivors n = 485 50–80 years old 3-20+ years post-diagnosis	Multiple logistic regression	Receipt of mammogram, usual source of care,^1^ recommendation by physician for mammogram and insurance coverage	Receipt of mammogram	Average
Andrykowski and Burris [45]	2010	USA	SEER database Breast cancer survivors n = 42 Colorectal cancer survivors n = 33 Hematological cancer survivors n = 38 1–5 years post-diagnosis Aged 25–75 years old	Multiple regression	Socio-demographics, cancer characteristics, mental health resource questionnaire	Use of formal and informal mental health services	Very good
Boehmer et al. [34]	2010	USA	Colorectal cancer survivors Aged 22–92 years old n = 253	Cox proportional hazard models	Colonoscopies, sigmoidoscopy, cancer type, stage, co-morbidities, outpatient visits, socio-demographics	Receipt of colorectal surveillance procedures	Very good
Cooper et al. [29]	2000	USA	SEER-MEDICARE database Colorectal cancer survivors Localised disease Surgically treated >65 years old n = 5, 716	Chi-square test	Socio-demographics, inpatient claims, outpatient claims, use of endoscopic procedures (colonoscopy, polypectomy or biopsy)	Receipt of colorectal surveillance procedures	Very good
Cooper and Payes [28]	2006	USA	SEER-MEDICARE database Colorectal cancer survivors >65 years old n = 62, 882 survived 1 year follow-up n = 35, 784 survived 3 year follow-up	Logistic regression	Medicare claims^2^ for colonoscopy, sigmoidoscopy or barium enema, co-morbidities	Use of surveillance procedures for colorectal cancer within 3 years of diagnosis	Very good
Cooper, Kou and Reynolds [31]	2008	USA	SEER database Colorectal cancer survivors >65 years old n = 9, 426	Multivariate regression	Number of physician visits, receipt of carcino-embryonic antigen blood test (CEA),^3^ colonoscopy, CT and PET scans	Adherence to guidelines for cancer follow-up	Good
Doubeni et al. [27]	2006	USA	Breast cancer survivors n = 797 at baseline (end of treatment) n = 262 after 5 yrs >55 years old 4 geographically diverse Health Maintenance Organisations (HMOs).^4^	Generalised estimated equations (GEE)	Receipt of mammograms. age, date and stage at/of diagnosis, treatment. co-morbidities. visits to primary care provider (primary care physician) and outpatient visits	Receipt of yearly mammogram and visits to physicians	Very good
Earle et al. [23]	2003	USA	SEER database Breast cancer survivors > 65 years old, n = 5,965 Controls n = 6,062	Multivariate regression	Frequency of visits to primary care physician, oncologists, other and teaching hospitals, receipt of flu vaccine, lipid test, cervical exam, colon exam, bone densitometry and diabetes test	Visits to physicians and receipt of preventive medicine	Very good
Earle and Neville [19]	2004	USA	SEER database Colorectal cancer survivors > 65 years old n = 14,884	Logistic regression	Co-morbidities, socio-demographics, receipt of flu vaccine, lipid testing, bone densitometry and cervical screening	Visits to physicians and receipt of preventive medicine	Very good
Earle, Neville and Fletcher [43]	2007	USA	Breast, lymphoma, colorectal, melanoma and other cancer survivors Mean age 60 years n = 1,111 Controls n = 4,444	Logistic regression `	Mental health diagnoses, co-morbidities, socio-demographics, use of primary care physician, oncologist, psychiatrists, psychologists, social workers and inpatient hospitalisations (both general and mental).	Use of mental health provider services	Good
Ellison et al. [33]	2003	USA	SEER database Colorectal cancer survivors >65 years old n = 52, 105	Kaplan-Meier survival analysis Unconditional regression analysis Cox regression	Socio-demographic, hospital and clinical characteristics, receipt of colonoscopy, sigmoidoscopy, endoscopy and barium enema	Differential receipt of colonoscopy, sigmoidoscopy, endoscopy and barium enema by race	Good
Gray et al. [41]	2000	Canada	Breast cancer survivors n = 731 Histologically confirmed and invasive	Stepwise logistic regression	Use of specialised supportive care services, wish to use services that were not accessed, social and demographic characteristics.	Use of professional supportive care services provided by the Ontario health care system	Very good
Gray et al. [42]	2002	Canada	Breast cancer survivors 63 % <60 years old 23–36 months post-diagnosis n = 731	Logistic regression	Supportive care from physicians and nurses, socio-demographics, illness and treatment information	Use of professional supportive care	Good
Grunfeld et al. [16]	1999	UK	Breast cancer survivors n = 148 Two district general hospitals	Two-tailed *t*-test and chi-square	Record of visits, average cost of visits, out-of patient expenses, waiting times, lost earnings and lost earnings of accompanying person	GP follow-up vs. Hospital follow-up. Cost-effectiveness and cost to patient,	Average
Grunfeld et al. [17]	2011	Canada	Breast cancer survivors n = 408 Nine tertiary cancer centres	Two-tailed *t*-test	Use of survivorship care plans (vs. no survivorship care plans) in primary care physician led follow-up. Frequency of visits to oncologists.	Primary care physician led follow-up	Very good
Keating et al. [25]	2006	USA	SEER-MEDICARE database Breast cancer survivors Stage 1 or 2 Underwent surgery >65 years old	Repeated-measures logistic regression	Mammogram receipt, visits to primary care physician medical oncologist, general surgeon, radiation oncologist and other specialists, socio-demographics	Factors related to mammography use	Very good
Keating et al. [11]	2007	USA	SEER database Breast cancer survivors >65 years old n = 37,967 in year 1 n = 30,406 in year 2 n = 23,016 in year 3	Repeated-measures logistic regression	Receipt of bone scans, tumour antigen tests (TAT), Chest x-rays and other abdominal/chest imaging, frequency of visits to physicians and socio-demographics	Receipt of a number of surveillance procedures and visits to physicians over time	Very good
Khan et al. [38]	2010	UK	GPRD database Breast cancer survivors N = 18, 612 Colorectal cancer survivors N = 5, 764 Prostate cancer survivors N = 4, 868 >30 years old 5 years post-diagnosis Controls N = 116,418	Multivariate regression	Socio-demographics, use of primary care, frequency of visits	Primary care consultations	Very good
Khan, Watson and Rose [20]	2011	UK	GPRD database Prostate cancer survivors N = 4,868 Breast cancer survivors N = 18,612 Colorectal cancer survivors N = 5,764 Controls N = 145,662	Logistic regression	Co-morbidities, screening (PSA, cervical, mammogram), receipt of preventative procedures and socio-demographics	Receipt of screening and preventative care	Very good
Knopf et al. [37]	2001	USA	SEER database Colorectal cancer survivors >65 years old n = 52, 283	Kaplan-Meier survival analysis	Receipt of colonoscopy, sigmoidoscopy, endoscopy and barium enema, age, tumour stage at diagnosis and year of diagnosis	Receipt of bowel surveillance procedures	Very Good
Lafata et al. [30]	2001	USA	Colorectal cancer survivors n = 251	Kaplan-Meier survival analysis Cox proportional hazards	Socio-demographics, receipt of colonoscopy, CEA, barium enema, chest x-ray, MRI’s, ultrasounds and liver analysis	Receipt of colon screening procedures and other procedures	Very good
Mahboubi et al. [15]	2007	France	Colorectal cancer survivors <65 years old N = 389	Logistic regression	Co-morbidities, chest radiograph, abdominal ultrasound, colonoscopy, CT, TAT, blood tests and reason for testing (routine or symptomatic)	Characteristics associated with visits to GPs	Very good
Mandelblatt et al. [13]	2006	USA	Breast cancer survivors n = 418 Stage 1 and 2	Multivariate linear regression	Calendar diary of health service use, socio-demographics, cancer treatment information, co-morbidities and psychological status survey	Patterns and determinants of health service use	Very good
Mayer et al. [35]	2007	USA	NCI 2003 HINTS^5^ n = 619 Breast cancer survivors n = 119 Prostate cancer survivors n = 62 Colorectal cancer survivors n = 49 Others n = 389	Logistic regression	Based on the health belief model (HBM),^6^ cancer communication, cancer history, general cancer knowledge, cancer risk and screening, health status and demographics.	Screening practices and beliefs	Very good
McBean, Yu and Virnig [39]	2008	USA	SEER database: Uterine cancer survivors >65 years old n = 14,575 Controls n = 58,420	Multivariate logistic regression Generalised equation modelling	Receipt of flu vaccine, bone densitometry, colorectal screening and mammogram no. of physician services and socio-demographics	Use of preventive services and frequency of physician visits	Very good
Mols, Helfenrath and van de Poll-Fanse [14]	2007^a^	Netherlands	Endometrial cancer Prostate cancer Non-Hodgkin’s lymphoma survivors n = 1,112	Linear regression Multivariate linear regression	SF-36, self-reported health service use, frequency of visits, co-morbidities and socio-demographics	Patterns of physician use	Very good
Mols, Coebergh and van de Poll-Fanse [22]	2007^b^	Netherlands	Endometrial cancer Prostate cancer, Hodgkin’s and non-Hodgkin’s lymphoma survivors n = 1,231	Chi-square and multivariate logistic regression	Co-morbidity, socio-demographics, use of medical specialist, general practitioner, additional services (physiotherapist. and psychologist)	Frequency of physician use	Very good
Oleske et al. [47]	2004	USA	Breast cancer survivors Aged between 21–65 years n = 123	Multivariate logistic regression	Use and frequency of physician and admissions, services in past 12 months. reasons for hospitalisations, SRS (social responsiveness scale) and CES-D (depression scale)	Determination of factors associated with hospitalisation	Very good
Peuckmann et al. [12]	2009	Denmark	Breast cancer survivors n = 1,316 Controls n = 4,865	Risk ratios and multiple logistic regression analysis	Frequency of physical visits, socio-demographics, physical activity and BMI. HR-QOL (SF-36) and chronic pain	Frequency and determinants of health service use	Very good
Schapira, McAuliffe and Nattinger [32]	2000	USA	SEER database Breast cancer survivors >65 years old n = 3,885	Logistic model	Receipt of mammogram, co-morbidity, socio-economic status (SES) and preventive treatment received	Receipt of Mammogram over two year period	Good
Schootman et al. [44]	2008	USA	SEER database Breast cancer survivors >65 years old n = 47, 643	Restricted iterative generalised least squares and first-order marginal quasi-likelihood estimation analysis	Frequency of Ambulatory-Care-Sensitive Hospitalizations (ACSH)^7^ SES, co-morbidity, demographics, availability of medical care, visits to primary care physician and oncologists	Frequency of Ambulatory-Care-Sensitive Hospitalizations	Very good
Simpson, Carlson and Trew [18]	2001	USA	Breast cancer survivors Time point 1 n = 46 Time point 4 n = 30 Controls Time point 1 n = 43 Time point 4 n = 25	ANOVA	Average cost of care, no. of cancer centre visits and a number of psychological distress indicators including BDI, POMS and Mental adjustment to cancer scale	Billing of Health care as a proxy to use. Visits to cancer centre Correlation of billing to distress.	Good
Snyder et al. [9]	2008^a^	USA	SEER database Colorectal cancer survivors >65 years old n = 1,541	Poisson regression and logistic regression	Clinical and socio-demographic characteristics, visits to primary care physician, oncologist or other physicians. Receipt of influenza vaccine, cholesterol screening, mammogram, cervical screening and bone densitometry	Frequency of physician visits and receipt of preventive care	Very good
Snyder et al. [10]	2008^b^	USA	SEER database Colorectal cancer survivors >65 years old n = 20,068	Poisson regression and logistic regression analysis	Co-morbidities, socio-demographics, visits to primary care physician, oncologist and other physicians, receipt of influenza vaccine, cholesterol screening, mammogram, and bone densitometry	Visits to physicians and receipt of preventive care	Good
Snyder et al. [24]	2009^a^	USA	SEER database Breast cancer survivors >65 years old n = 23, 73 Controls n = 23, 731	Poisson regression and logistic regression analysis	Use of physician and oncology services, receipt of 5 preventive care services and socio-demographics.	Visits to physicians and oncologists and preventive medicine	Good
Snyder et al. [26]	2009^b^	USA	SEER database Breast cancer survivors >65 years old Stages 1–3 n = 1,961 Controls n = 1,961	Poisson regression and logistic regression analysis	Co-morbidities, clinical and demographic characteristics, visits to primary care physician, oncologists and other physicians	Frequency of visits to physicians	Good
Van de Poll-Fanse et al. [21]	2006	Netherlands	Breast cancer survivors Invasive n = 183	Logistic regression	Co-morbidities, spontaneously reported problems, use of GP, medical specialist and physiotherapist, health status and psychological well-being	Use of physician services	Good
Yu, McBean and Virnig [40]	2007	USA	SEER database Colorectal cancer survivors >65 years old n = 112, 737.	Logistic regression and poisson regression	Socio-demographic characteristics, co-morbidities, receipt of mammogram, visits to primary care physician, Gynaecologists only, oncologists and other	Receipt of mammogram and visits to physicians	Good

^1^Usual source of care refers to whether an individual receives care from the same physician or different physicians; ^2^Medicare is a government-funded medical care plan in USA, whereby individuals aged 65 and over that covers medical expenses such as doctor's visits, hospital stays, drugs and other treatment; ^3^CEA testing is used as a tumour marker for particular cancers, such as colorectal; ^4^HMOs provide their members with medical services for a fixed fee; ^5^NCI HINTS is the Health Information National Trends Survey, which collects nationally represented information on how the American public find and use information on cancer; ^6^Developed by Hochbaum (1958) is an explanatory and predictive model of health behaviours and includes attitudes and beliefs of an individual; ^7^ACSH are hospitalizations which could have been prevented if primary care services had been initially accessed by the individual.

**Table 2:** Study results

**Author**	**Outcome**	**Predisposing characteristics**	**Enabling characteristics**	**Need characteristics**
Andersen and Urban [36]	Follow-up cancer surveillance		Previous diagnosis via this method. Physician recommendation.	70 % received mammography in first year. 72 % received mammography in two years.
Andrykowski and Burris [45]	Mental health service use		Rural are less likely to have mental health services within 30 mile radius.	18 % of non-rural and 8 % of rural CSs utilised psychologist services.
Boehmer et al. [34]	Follow-up cancer surveillance	Female CSs less likely than male CSs to receive either colonoscopy or sigmoidoscopy within 1 and 3 years of treatment. Black CSs more likely than white CSs to receive follow-up screening.	A greater number of outpatient visits.	
Cooper et al. [29]	Follow-up cancer surveillance	Older CSs less likely than younger CSs to receive screening within 5 years of diagnosis.	Geographical variation in receipt.	CSs with a co-morbidity were less likely than CSs without a co-morbidity to receive colonoscopy or sigmoidoscopy in first year of survivorship. Increase in receipt of surveillance procedures over time. Over a 3 year period: 58 % of CSs received on average 2.8 colonoscopies; 19 % received on average 2.0 colonoscopies.
Cooper and Payes [28]	Follow-up cancer surveillance	Older CSs less likely than younger CSs to receive screening within 3 years of diagnosis. Female CSs were more likely than male CSs to receive screening within 3 years of diagnosis. White CSs were more likely than black CSs to receive screening.	Visits to a primary care physician.	Receipt of colonoscopy increased over time. No difference in receipt of FOBT or colonoscopy between CSs and controls.
Cooper, Kou and Reynolds [31]	Follow-up cancer surveillance	Older CSs less likely than younger CSs to receive follow-up which adheres to professional guidelines. White CSs more likely than Black CSs to receive follow-up which adheres to professional guidelines.		CSs with a comorbidity were more likely than CSs without a co-morbidity to receive CEA testing. CSs with later stage and undifferentiated tumour were more likely to exceed guidelines. Decrease over time in receipt of barium enema and sigmoidoscopy.
Doubeni et al. [27]	Primary care use Follow-up cancer surveillance	Younger CSs were more likely to receive a mammography compared to older CSs. White CSs were more likely to receive a mammography compared to black CSs.		Visits to a family physician increased from 55-71 % over a 5 year period. CSs with co-morbidities were less likely than CSs without co-morbidities to receive a mammography.
Earle et al. [23]	Primary care use Preventative care	Older CSs were less likely to receive preventative care compared to younger CSs. Black CSs were less likely to receive preventative care compared to white CSs CSs with lower SES were less likely to receive preventative care compared to CSs with higher SES. CSs residing in a rural area were less likely to receive preventative care compared to CSs residing in an urban area.	Visits to a primary care physician and an oncology specialist.	52 % of CSs followed up by both an oncology specialist and primary care physician. 41 % of CSs followed up by primary care physician only. 4 % of CSs followed up by oncology specialist only. CSs with a co-morbidity were more likely to receive preventative care compared to CSs without a co-morbidity. CSs received more preventative care compared to controls.
Earle and Neville [19]	Primary care use Preventative care	Non-white CSs were less likely than white CSs to receive preventative care. Older CSs compared to younger CSs were less likely to receive preventative care.	No visits to primary care physician or oncology specialist led to less preventative care receipt.	CSs compared to general population were more likely to visit a primary care physician. 50 % of CSs visited oncology specialist and other physicians. 8 % of CSs visited oncology specialist only. CSs with a co-morbidity were less likely to receive lipid testing than CSs without a co-morbidity. CSs were less likely than controls to receive lipid or cholesterol testing.
Earle, Neville and Fletcher [43]	Mental health service use	Younger breast CSs (>65 years old) were most likely to use mental health services.		CSs compared to controls were more likely to report anxiety and sleep disorders and have greater use of mental health services. 18 % of CSs made at least 2 or 3 visits to a psychologist. Breast cancer survivors had greatest level of use.
Ellison et al. [33]	Follow-up cancer surveillance	White CSs were more likely to receive post-treatment surveillance compared to black cancer survivors.		Use of colorectal surveillance test increased over time for colorectal CSs.
Gray et al. [41]	Mental health service use	Younger CSs were more likely to use mental health services than older CSs. CSs who were employed were more likely to receive mental health services than CSs who were unemployed. CSs who were students were more likely to receive mental health services than CSs who were not students.	CSs who had additional health insurance were more likely to use mental health services than CSs who did not have additional insurance.	
Gray et al. [42]	Mental health service use	Younger CSs were more likely to use mental health services compared to older survivors.	CSs with additional health insurance, higher income and higher education were more likely to use mental health services compared to CSs without additional health insurance, with lower income and education.	Younger CSs, with additional health insurance and a higher level of education expressed a need for services that they were not receiving. 31 % CSs made at least one visit to a mental health professional, 5 % to a psychologist and 4 % were to a psychiatrist. 0-11 % of CSs used social services, dieticians, physiotherapists and other health care providers.
Grunfeld et al. [16]	Hospital care			CSs led by hospital follow-up had lower health service use compared to CSs led by primary care physician follow-up.
Grunfeld et al. [17]	Primary care use		A small proportion of CSs followed up by primary care physician made contact with an oncologist in a 12 month period.	
Keating et al. [25]	Primary care use Follow-up cancer surveillance	Younger and white CSs were more likely to receive a mammogram than CSs who were older and black.	Visits to oncology specialists led to a greater likelihood in the receipt of mammogram by CSs.	Visits to primary care physicians increased over time, whereas visits to oncology specialists decreased over time. A recent diagnosis, a second cancer, large tumour and no radiotherapy receipt led to a greater likelihood of mammography receipt.
Keating et al. [11]	Primary care use	Younger CSs were more likely to visit an oncology specialist.		The role of care provided by both primary care physicians and oncology specialists decreased over a three year period. Annual follow-up was provided to 51 % of breast CSs by primary care physicians and 27 % of CSs by oncology specialists.
Khan et al. [38]	Follow-up cancer surveillance Preventative care	Older CSs were more likely than younger survivors to receive influenza vaccination.	A greater number of visits to a health care provider facilitated receipt of preventative care.	Receipt of mammography decreased over time. CSs compared to the general population had similar rates of cholesterol testing and blood pressure monitoring. Colorectal CSs were more likely to receive PSA testing. Breast CSs were less likely than the general population to receive preventative care with the exception of bone densitometry.
Khan, Watson and Rose [20]	Primary care use			Visits to primary care physician increased over time by CSs. CSs compared to the general population were more likely to visit their primary care physician.
Knopf et al. [37]	Follow-up cancer surveillance			Receipt of a number of colorectal cancer surveillance procedures increased over time for colorectal CSs following treatment.
Lafata et al. [30]	Follow-up cancer surveillance	Older CSs were less likely than younger CSs to receive follow-up screening within 5 years of treatment with curative intent. White CSs were more likely to receive follow-up screening than black CSs.		Receipt of colonoscopy and CEA and metastatic disease testing increased over time.
Mahboubi et al. [15]	Primary care use Follow-up cancer surveillance	CSs living in specific geographic areas.	21 % of all colorectal surveillance procedures within 3 years of curative surgery were delivered by a primary care physician and 41 % by a gastroenterologist or oncology specialist.	Increased visits to primary care physicians over time.
Mandelblatt et al. [13]	Primary care use Hospital care Follow-up cancer surveillance	White CSs were more likely to utilise health services than black CSs.		CSs with a co-morbidity, self-reported poor functioning and high depression scores had greater use and cost of health services. Within the first year of survivorship an average of 14 visits per CS was made to a medical provider. An average of 3 visits to a physiotherapist/occupational therapist per CS was made. 62 % of CSs received a mammography.
Mayer et al. [35]	Follow-up cancer surveillance	CSs had a greater absolute or comparative risk of developing cancer compared to the general population.	Physician recommendation increased likelihood of screening.	Greater receipt of screening among CSs compared to general population.
McBean, Yu and Virnig [39]	Preventative care	Older and black CSs were less likely to receive preventative care compared to younger and white CSs.	Uterine CSs most likely to receive mammography if seen by a gynaecologist or an oncology specialist. CSs most likely to receive bone densitometry and influenza vaccination if seen by a primary care physician. Receipt of each test most likely if at least 5 visits to a physician and no overnight hospital stays.	Uterine CSs more likely to receive colorectal or breast cancer screening than the general population.
Mols, Helfenrath and van de Poll-Fanse [14]	Primary care use Hospital care			CSs had similar use of primary care physician compared to general population. 0-11 % utilised social services, dieticians and physiotherapists.
Mols, Coebergh and van de Poll-Fanse [22]	Primary care use Mental health service use	CSs diagnosed between 10 and 15 years previously, who were single or divorced were less likely to utilise health services compared to CSs diagnosed at different time-points and CSs with partners.	Higher education enabled use of mental health services.	CSs with a co-morbidity were twice as likely to utilise primary care physician services than CSs without a co-morbidity. Endometrial CSs had greater use of health services than the general population. 1-10 % of CSs utilised psychologist services.
Oleske et al. [47]	Hospital care			25 % of CSs had at least one overnight hospital stay. Experiencing menopausal symptoms and high CES-D scores led to more inpatient stays.
Peuckmann et al. [12]	Primary care use	Older CSs (<75 years old) were most likely to visit their primary care physician within 3 years of treatment.		CSs had similar primary care physician use compared to the general population. Breast CSs had greater use of allied health professionals than the general population.
Schapira, McAuliffe and Nattinger [32]	Follow-up cancer surveillance			CSs with a co-morbidity were less likely than CSs without a co-morbidity to receive a mammography. 23 % of CSs received a macmography in the first 2 years following treatment.
Schootman et al. [44]	Hospital care	Older, divorced or widowed CSs were more likely to be an inpatient than CSs who were younger, not divorced and not widowed. CSs who were not black or white were less likely to be an inpatient than CSs who were black or white.	CSs living in an impoverished area were more likely to have an overnight stay in hospital compared to CSs living in more affluent areas. CSs who had visited their physician at least once were less likely to have an overnight stay than CSs who did not visit their physician.	13 % of CSs had at least one overnight hospital stay. CSs with at least one co-morbidity were more likely to have an overnight stay compared to CSs without a co-morbidity.
Simpson, Carlson and Trew [18]	Primary care		Participation in psychotherapy intervention led to a reduction in health service use by CSs.	
Snyder et al. [9]	Primary care use Preventative care	Younger, female colorectal CSs were more likely to receive care form both a primary care physician and oncology specialist compared to older, male CSs. Older CSs less likely to receive cholesterol testing, cervical examination and bone densitometry than younger CSs.	CSs who lived in an urban area compared to CSs who lived in a rural area were more likely to receive mammography, cervical smear and influenza vaccination. Most likely to receive preventative care if followed-up by both primary care physician and oncology specialist. CSs living in rural areas were less likely to receive mammography compared to CSs living in urban areas.	CSs had increased visits over time to primary care physician. CSs had decreased visits to oncology specialists over time. Receipt of mammography and cervical screening decreased over time. Bone densitometry remained low. Rates of influenza vaccination fluctuated over time. CSs with a co-morbidity were less likely to receive cervical screening and bone densitometry, but greater receipt of influenza vaccination, cholesterol testing than CSs without a co-morbidity.
Snyder et al. [10]	Primary care use Preventative care	Older CSs (>85 years old) were more likely to receive care from a primary care physician compared to CSs aged <75 years old. Black CSs were more likely to receive care from physicians other than a primary care physician. Black CSs compared to white CSs were less likely to receive care from a primary care physician. Non-white CSs were less likely to receive influenza vaccination than white CSs. Older CSs less likely to receive cholesterol testing and bone densitometry but were more likely to receive influenza vaccination than younger CSs.	Most likely to receive preventative care if followed-up by both primary care physician and oncology specialist.	CSs had increased visits over time to other physicians. CSs with a co-morbidity were less likely to receive cervical screening and bone densitometry, but greater receipt of influenza vaccination, cholesterol testing and mammography than CSs without a co-morbidity.
Snyder et al. [24]	Primary care use Follow-up cancer surveillance Preventative care		Breast CSs were most likely to receive preventative care if visits were made to an oncology specialist and a primary care physician.	Majority of CSs followed up by both oncology specialist and primary care physician over time. Increased visits to oncology specialist over time. Decreased visits to primary care physician over time. Breast CSs had greater use of mammography compared to the general population. Breast CSs received less preventative care than the general population. CSs more likely to receive preventative care if general population has a co-morbidity.
Snyder et al. [26]	Primary care use Preventative care			Increased visits to primary care physician over time. Decreased visits to oncology specialist over time. Breast CSs received less preventative care than the general population.
Van de Poll-Fanse et al. [21]	Primary care use	Younger CSs were more likely to visit an oncology specialist compared to older CSs.		Breast CSs had similar primary care physician use as the general population.
Yu, McBean and Virnig [40]	Follow-up cancer surveillance	Older CSs were less likely to receive mammography compared to younger CSs.	CSs with state health insurance were less to receive a mammography compared to CSs with alternative health insurance. CSs living in a rural area were less likely to receive mammography compared to CSs living in an urban area. Care from a gynaecologist rather than a primary care physician led to greater receipt of mammography.	

### Primary care

#### Predisposing characteristics

The US health-care system permits individuals to choose to an extent which health care provider provides their care in relation to their insurance plan restrictions or absence of an insurance plan. Specialists are included in this section on primary care as data indicated that there is a limited set of services used by patients in the US health care system that may be provided by a specialist such as an oncologist or by a primary care physician. There was a consistent association between contact with primary care and predisposing characteristics such as age, ethnicity, gender and time since diagnosis. Contact with services for cancer-related problems and all symptoms and illnesses are included within the results. Studies were inconsistent regarding whether younger or older CSs were more likely to visit an oncologist or a primary care physician. Younger colorectal CSs were more likely to visit both a primary care physician and an oncologist, whereas older colorectal CSs were more likely to visit only a primary care physician [9,10]. Moreover, younger breast CSs from the US were more likely to visit an oncologist [11], whereas older breast CSs from Denmark were most likely to visit their primary care physician [12]. Differences in the pattern of primary care utilisation by ethnicity were found across cancer sites. Black colorectal CSs were more likely to receive care from physicians other than a primary care physician, whereas, white colorectal CSs were more likely to receive care from a primary care physician [10]. Furthermore, white breast CSs were more likely to utilise health services compared to black CSs [13]. Compared to male colorectal CSs, female colorectal CSs were more likely to receive care from a primary care physician and an oncologist than either one of these physicians only [9]. Only one study (conducted in the Netherlands) addressed time since diagnosis and the use of primary care services. Dutch endometrial CSs diagnosed between 10 and 15 years previously were less likely to visit a primary care physician than CSs diagnosed within 10 years or very long-term survivors at least 15 years post-diagnosis [14].

#### Enabling characteristics

Area of residence, participation in an intervention, primary care physician-orientated follow-up and marital status (as a proxy for social support) acted as significant enablers for primary care service use. French colorectal CSs from the Saône and Loire regions were more likely to have made regular contact with a primary care physician than CSs from other French regions [15]. One UK study demonstrated that the orientation of follow-up cancer care was important for health service use. CSs who received hospital-oriented follow-up had lower health-care utilisation rates compared to CSs whose follow-up care was oriented by their primary care physician [16]. A small proportion of Canadian breast cancer survivors whose follow-up care was provided by a primary care provider made visits to an oncologist over a 12 month period [17]. A reduction in health service use was observed for breast CSs who participated in a psychotherapy-based intervention to reduce stress [18]. One study from the Netherlands found that a lack of social support (i.e. CSs who were single or divorced) led to less use of health services compared to CSs who were married [14].

#### Need characteristics

A number of studies found that co-morbidities were associated with visits to primary care. Breast CSs with a co-morbidity, or self-reported poor functioning or high depressive mood had greater service utilisation and health-care costs than breast CSs without a co-morbidity, or who reported better functioning or had low scores low depressive mood [13]. Similarly in the Netherlands health service utilisation rates were two times greater for cancer survivors with a co-morbidity compared to CSs without a co-morbidity [14]. It is not clear from the findings whether CSs were more or less likely to utilise primary care services compared to a non-cancer control population. Compared to individuals without cancer, US and UK colorectal CSs, UK prostate CSs and Dutch endometrial survivors [14,19,20], were significantly more likely to visit their primary care physician. However, Dutch CSs and Danish breast CSs had similar primary care physician use as non-cancer controls [12,21,22]. In terms of the level of contact made with primary care patterns of utilisation changed over time. Contact with oncologists largely decreased over time, whereas visits to a primary care physician largely increased over time. Annual visits to primary care physicians and oncologists were made by 51 % and 27 % of breast CSs respectively [11]. Within the first year of survivorship there was a high health service utilisation rate for breast CSs; an average of 14 visits per individual was made to a medical provider including a primary care physician or an oncologist [13]. Approximately 50 % of CSs visited an oncologist alongside other physicians, whereas 8 % of CSs made visits to only an oncologist [19]. One cross-sectional study reported that 52 % of breast CSs made visits to both an oncologist and a primary care physician, whereas 41 % visited a primary care physician only and 4 % visited an oncologist only [23]. The level of contact made to both primary care physicians and oncologists decreased over a three year period, from 70-42 % and 30-17 %, respectively. One longitudinal study found that in the first year of follow-up, the majority of breast CSs made visits to both physicians; over time the number of visits made to a primary care physician decreased and the number of visits made to an oncologist increased [24]. In contrast, further studies reported an increase in the number of visits to a primary care physician and a decrease of visits to an oncologist and other physicians over time for colorectal and breast CSs respectively [9,10,13,20,25-27].

### Follow-up cancer surveillance

#### Predisposing characteristics

Follow-up cancer surveillance includes any test which is used to screen for recurrence or metastases of the primary cancer. Each study which addressed the predisposing characteristics associated with CSs who utilised follow-up cancer surveillance procedures was conducted in the USA. Predisposing characteristics found to be significantly related to follow-up cancer surveillance were age, ethnicity, gender and health beliefs. Older age was associated with receipt of less follow-up cancer surveillance procedures. Compared to their younger counterparts, older colorectal CSs were consistently less likely to receive surveillance procedures within 3 years of diagnosis [28], 5 years of diagnosis and treatment [29,30], and they were also less likely to receive follow-up surveillance in adherence to government follow-up guidelines [31]. At the time of the study a number of guidelines for colorectal cancer follow-up care had been developed but none had been widely implemented. Therefore, the authors amalgamated these guidelines to create a minimum number of service and procedure receipt recommendations. These recommendations included: at least two visits to a physician per year; the receipt of at least two Carcino-Embryonic Antigen (CEA) tests within each of the first two years of survivorship; and the receipt of at least one colonoscopy within the first three years of survivorship. Excess of government guidelines included receiving the minimum level of care in addition to the receipt of at least one CT scan and/or at least one PET scan [31]. Older breast CSs were also less likely to receive mammograms compared to their younger counterparts [25,27,32].

The majority of the evidence regarding ethnicity and uptake of follow-up surveillance found that white CSs were more likely to receive follow-up screening [25,27,28,30,32,33] and to adhere to the guidelines specified above [31] than CSs of other ethnicities. A study based at a ‘safety-net hospital’ which provided care to underserved populations such as ethnic minorities found that black colorectal CSs were more likely to receive follow-up colonoscopies within 3-years of curative resection than CSs of other ethnicities [34]. The extent to which gender predisposes follow-up cancer surveillance is unclear. Within the first three years of diagnosis, females were more likely to utilise colorectal screening compared to male colorectal CSs, [28] but were less likely than males to receive either colonoscopy or sigmoidoscopy within 1 and 3 years of treatment [34]. Colorectal CSs perceived a greater absolute and comparative risk for developing cancer leading to greater receipt of screening compared to non-cancer controls [35].

#### Enabling characteristics

Factors which enabled receipt of follow-up surveillance included visits to specific health-care providers, frequency of health-care contact, area of residence, physician recommendation of test and previous cancer diagnosis detected via screening procedure. Variations in colorectal cancer surveillance uptake by French colorectal CSs were dependent on type of physician; 21 % of all colorectal surveillance procedures within 3 years of curative surgery were delivered by a primary care physician and 41 % by a gastroenterologist or an oncologist [15]. An increased number of outpatient visits led to a greater likelihood of receiving colonoscopy or sigmoidoscopy within 3 years of treatment for colorectal CSs [34]. A significant geographical variation in receipt of surveillance procedures was also observed for colorectal CSs [29]. An explanation for this variation given by the authors was the influence of local practice on testing. Moreover, a physician recommendation of follow-up procedures increased the likelihood of procedure uptake for breast CSs and prostate CSs [35,36]. Receiving a previous breast cancer diagnosis detected by a mammogram was significantly and positively associated with subsequent receipt of mammogram in the survivorship period [36].

#### Need characteristics

Co-morbid illnesses, cancer stage and treatment history were significant need characteristics associated with receipt of follow-up surveillance. Presence of a co-morbid illness led to a lower likelihood of receiving follow-up surveillance. Colorectal CSs with a co-morbidity were less likely to receive colonoscopy and sigmoidoscopy than CSs without a co-morbidity in the first year of survivorship and were less likely to receive CEA testing to the standard recommended by US follow-up guidelines which included receiving at least two CEA tests per year [29,31]. This finding is further supported by the breast cancer survivorship literature. Breast CSs with co-morbidities compared to CSs without co-morbidities were less likely to receive a mammogram [25,27,32]. Colorectal CSs with later stage and an undifferentiated cancer were more likely to exceed recommended guidelines which included receipt of at least one CT and/or PET scan, in addition to minimum recommendations [31]. Further factors associated with mammography receipt for breast CSs were a more recent diagnosis, a secondary cancer, a large tumour and no history of adjuvant radiotherapy [11]. Comparisons of surveillance procedure receipt between CSs and non-cancer controls were largely consistent. Breast CSs had significantly greater use of mammogram compared to controls when adjustments were made for age, race and access to health-care [26,35]. A similar result was found for PSA testing among prostate CSs [35]. However, one study found that having a cancer history did not lead to differential receipt of Faecal Occult Blood Test (FOBT) or colonoscopy compared to the general population [28]. Rates of follow-up surveillance receipt varied across studies and cancer sites, overall rates of uptake were low to moderate. Eleven percent of colorectal CSs received at least one surveillance procedure each year [28]. Over a three year period, 58 % of colorectal CSs in the USA received on average 2.8 colonoscopies, a lower percentage (19 %) received on average 2.0 sigmoidoscopies and there was an observed decrease in the receipt of barium enema and sigmoidoscopy [29,31]. In contrast to this finding some studies reported an increase over time in the receipt of a number of colorectal cancer surveillance procedures following treatment specifically colonoscopy, CEA testing and metastatic disease testing [28-31,37].

For breast CSs the receipt of mammography decreased over time, with the exception of one study. Sixty-two percent of breast CSs received a mammography in both the first and second years of survivorship whereas 23 % of breast CSs received a mammogram in either year of the first two years of survivorship [13,32]. A further two studies reported a decrease over time in mammogram receipt [27,38] whereas another study reported an increase over a two year period [36]. A few studies assessed the level of surveillance receipt in comparison to government-recommended guidelines. The majority of CSs did not meet recommended levels of surveillance receipt; in one study 17 % of colorectal CSs met the guidelines, 23 % exceeded the guidelines and 60 % failed to meet the guidelines. Guidelines have been described above [31]. In the first year of survivorship between 11-59 % of breast CSs received surveillance procedures such as a chest x-ray which were not recommended by American Society of Clinical Oncology (ASCO) guidelines [11].

### Preventive care

#### Predisposing characteristics

Significant predisposing variables for receipt of preventive care included ethnicity and age. White CSs were consistently more likely to receive preventive care than non-white CSs. Colorectal CSs who were non-white were less likely to receive preventive care, particularly influenza vaccination [9,19] than white CSs. Furthermore, white breast and uterine CSs were more likely to receive preventive care compared to black CSs [23,39]. The evidence was consistent regarding age and receipt of preventive care, whereby older CSs were less likely to receive preventive care (with the exception of influenza vaccination) than their younger counterparts. Older colorectal CSs were less likely to receive preventive care including cholesterol testing, cervical examination, bone densitometry and mammography, [9,10,19,40] but were more likely to receive influenza vaccinations than younger cancer survivors [10]. Older breast and uterine CSs were less likely to receive preventive care compared to younger CSs [23,39]. A UK-based study found that colorectal, breast and prostate CSs over the age of 65 were more likely to receive influenza vaccination than younger CSs [38].

#### Enabling characteristics

Enabling characteristics associated with the receipt of preventive care included visits made to specific physicians, frequency of health-care contact, area of residence, socio-economic status, overnight hospitalisations and health insurance. Visits to more than one type of health-care provider (i.e. primary care physician and oncologist) were more likely to facilitate receipt of preventive care. This finding was consistent for both colorectal and breast CSs respectively [9,10,23,24,26]. Visits made by colorectal CSs to either a primary care physician or an oncologist facilitated receipt of preventive care, but not at the same level as visits made to both health-care providers; whereas CSs who did not visit either health care provider received the lowest levels of preventive care receipt [19]. Moreover, general preventive care (i.e. bone densitometry) was more likely to be delivered by a primary care physician than any other type of physician. Oncologists and gynaecologists were more likely to deliver cancer-related preventive care such as mammography to colorectal and uterine CSs respectively [9,40]. Moreover, an increasing number of visits to a health-care provider was the strongest predictor for receipt of preventive care for a sample of UK CSs [38]. A study of uterine CSs in the US quantified this amount as 5 or more visits to a physician [39]. Living in an urban area was significantly associated with greater receipt of mammography, influenza vaccination and cervical smear among colorectal CSs [9,40] and general preventive care among breast CSs [23]. One study reported that breast CSs who had lower socio-economic status were less likely to receive preventive care compared to breast CSs with higher socio-economic status [23]. Receipt of preventive health services among uterine CSs who had not been hospitalised was greater compared to CSs who had been hospitalised. Furthermore, colorectal CSs with private health insurance were more likely to receive a mammogram compared to colorectal CSs with government-funded health insurance [39].

#### Need characteristics

Colorectal CSs with a co-morbidity were less likely to receive lipid testing, cervical screening and bone densitometry, but were more likely to receive influenza vaccination, cholesterol testing and mammography compared to CSs without a co-morbidity [9,10,19]. Conversely, breast CSs with a co-morbidity were more likely to receive overall preventive care than CSs without a co-morbidity [23]. Evaluation and Management meetings refer to service contact which is not for the intention of procedures or tests. Survivors with a co-morbidity who attended for Evaluation and Management meetings were more likely to receive a mammography than CSs who did not attend for Evaluation and Management meetings [40]. Rates of preventive care receipt were not consistent between CSs and non-cancer controls. Breast and uterine CSs were more likely than controls to receive preventive care such as colorectal cancer screening [23,39]. An UK-based study found comparative rates of cholesterol testing and blood pressure monitoring between CSs (including prostate and breast cancers) and the general population, but a 19 % increased likelihood of PSA testing for colorectal CSs compared to the general population [38]. Comparisons of preventive care receipt between breast CSs and healthy controls demonstrated that CSs were less likely to receive preventive care, particularly lipid and cholesterol testing [19,24,26]. However, they were more likely to receive bone densitometry or general preventive care than non-cancer controls with co-morbidities [24,38]. Colorectal CSs receipt of mammography and cervical screening decreased over time, receipt of bone densitometry remained low, whereas rates of influenza vaccination fluctuated over time [9].

### Hospital care including mental health services

#### Predisposing characteristics

Support was found for age, employment and student status as predisposing characteristics associated with the use of mental health services. Younger age (<65 year olds vs. >65 year olds) was significantly associated with seeking mental health or supportive care services among breast CSs in two Canadian studies [41,42] and among survivors of breast, lymphoma, colorectal, melanoma and other cancers in one US study [43]. Breast CSs who reported that they were currently employed or a student were more likely to utilise professional supportive care services [41].

Two US studies reported on the patterns of inpatient hospitalisations among breast CSs. Only one of these studies addressed the predisposing characteristics associated with being hospitalised. Ambulatory-Care-Sensitive Hospitalisations or preventable hospitalisations were associated with older age, being widowed or divorced and lower likelihood was associated with being of an ethnicity other than white and black [44].

#### Enabling characteristics

Education level, household income, health insurance and residential area were enabling factors associated with the use of mental health services. CSs with a high level of education were more likely to utilise mental health services compared to CSs with a lower level of education; this was supported by a study of endometrial, prostate and lymphoma CSs in the Netherlands [14] and breast CSs in Canada [41,42]. Household income which is strongly associated with educational level was a significant enabling characteristic for mental health service utilisation; both Canadian studies found that higher household income resulted in greater likelihood of using mental health services [41,42]. Further results from these two studies found that additional health insurance compared to government-funded insurance was associated with increased mental health service use [41,42]. One US study addressed the impact of urban or rural residence on receipt of mental health services among breast, colorectal and haematological CSs and found no significant difference in service receipt despite rural CSs being less likely to have psychiatric services within 30 miles of their home [45].

Enabling characteristics associated with inpatient hospitalisations included socio-economic status of residential area and previous visits to a physician. CSs from an impoverished area compared to a more affluent area were more likely to experience an Ambulatory-Care-Sensitive Hospitalisation. However, if CSs had visited a physician recently the risk of being hospitalised was reduced [44].

#### Need characteristics

Experiencing a psychological disorder and expressing an explicit need for mental health services was associated with use of mental health or supportive care services. CSs reported a greater need for and use of mental health services compared to the general population without cancer, due to a higher prevalence of anxiety and sleep disorders [43]. Canadian breast CSs were asked if they were in need of, or could not access mental health services; breast CSs who were younger, had additional health insurance or a high level of education, were working or studying were more likely to report an explicit need for services [42]. Rates of mental health service utilisation were low among CSs ranging from 1 % to18%. Between 1 and 10 % of CSs in the Netherlands utilised psychology services; survivors of lymphoma had the greatest use of psychology services compared to survivors of endometrial and prostate cancers [14]. There were low utilisation rates of both psychiatric and psychology services among Canadian breast CSs (4 % and 5 % respectively) [41]. Eighteen percent of USA CSs made on average 2 or 3 visits to a mental health professional and breast CSs were the highest users of services [43]. Between-study variation in rates may be due to different modes of access to services across health care systems.

Need-related factors associated with inpatient hospitalisations included having at least one co-morbidity, experiencing menopausal symptoms and higher scores on the Centre for Epidemiology Studies-Depression scale (CES-D). Between 13 % and 25 % of breast CSs had at least one overnight hospital stay [44,46].

The use of other health services such as social services or dietetics ranged from 0-11 % of CSs [22,42]. Danish breast CSs had greater utilisation of allied health care professionals than the general population, [12] and US CSs had an average number of 3 visits per survivor to a physiotherapist or an occupational therapist [13].

## Discussion

The results of this review are consonant with the Andersen Behavioural Model of health service utilisation. Younger, white and employed or student CSs were more predisposed to access and receive health care. Individuals over 65 years old represent an at-risk group and are encouraged by their health-care provider to receive vaccination annually [47]. According to the results of this review, older CSs were more likely to receive influenza vaccination but not other types of care. This discrepancy between older and younger CSs in terms of receipt of preventive services may be explained partly by physicians making decisions about the utility of preventive care based on the life expectancy of CSs, [9,10,40] – this may also explain the increased risk regarding inpatient hospitalisations among elderly CSs. The role of incentive payments to primary care physicians may play a part in the differential patterns of preventive services utilisation. Ethnicity was examined within US studies only and various explanations relating to economic differences, differences in health-seeking behaviours, preferences for treatment or perceptions of post-treatment cancer surveillance may account for reported ethnic disparities [19,30,33].

Visits to primary care physicians appeared to enable the implementation of care recommendations for CSs. Other facilitating factors which were more pertinent in non-nationalized or universal health-care systems were additional health insurance, a higher education, income and living in an urban, affluent area. Many samples included CSs who had insurance (e.g. Medicare) and this factor may be related to a sense of self-efficacy in terms of seeking information and negotiating the health-care system [42]. Cancer survivors had greater or at least similar frequency of contact with primary care physicians compared to the general population without cancer. Many cancer survivors may experience long-term health problems or further ill-health following treatment which require further care or specialist care. In many health-care systems a primary care physician may act as a gatekeeper for access to specialist services thus accounting for greater frequency of visits among the cancer survivor population.

Needs for care were also related to co-morbidity, later stage or undifferentiated tumour and menopausal or depressive symptoms. Younger, employed or student CSs expressed a need for mental health services. Overall, as CSs survived longer post-diagnosis they used less cancer-oriented care, with the exception of screening.

Although the review amalgamated literature regarding core health services, it excluded some health services such as complementary and alternative medicine due to resource restraints (and their perceived non-mainstream position). Services like complementary and alternative medicine are becoming increasingly important for CSs and require research attention. Limitations of the review include uncertainty about generalisability of findings. There is a need to give consideration to the merits of conducting comparative health care system research (including health service research in non-USA countries), particularly given the differing role of the oncologist between health-care systems and the role of insurance in obtaining access to care in USA studies. Fifteen studies comprised a secondary analysis of the SEER-Medicare database which included individuals over the age of 65 years and excluded individuals covered by other insurance plans or no insurance plan. Moreover, individuals were limited by their insurance plan regarding access to physicians and entitlement to receive particular procedures. For example, there are additional charges for receipt of procedures such as colonoscopy [33]. A further issue for the review was the lack of generalisation of results to US cancer survivors without medical insurance and thus a primary care physician may not be the first point of contact for care for some individuals. The SEER-Medicare database also did not provide information regarding reasons for use of services (i.e. cancer-related follow-up or for another condition). None of the papers reported the reasons for health service contact and whilst CSs appeared to be using relevant health services, this did not equate to follow-up care. It may not have been appropriate to compare utilisation rates between CSs and ‘healthy’ individuals from the general population as they may also have chronic illnesses; this is an inherent limitation within the included primary studies. Unfortunately, none of the included studies examined the nature and extent of the coordination of different services provided for CSs and this needs to be empirically tested.

The Andersen Behavioural Model of health service utilisation was used as the structural framework to organise the review. Although it is limited in its scope regarding potentially important behavioural variables, it does take into account the health beliefs of an individual. Health beliefs were not extensively addressed by the studies in the review. Indeed, only one study looked at the perception of risk and only in respect of cancer recurrence. There is a need to give empirical attention to the role of research evidence-based behavioural and ‘cognitive-behavioural’ constructs in order to improve our understanding preventive procedure receipt and adoption of health promoting behaviours by cancer survivors [48]. Future research efforts to understand health service use by cancer survivors should consider supplementing or expanding the Andersen Behavioural Model to include behavioural and cognitive components (e.g. subjective norms) from models such as the Theory of Planned Behaviour [49].

## Conclusions

The emergence and evaluation of practice guidelines over time may impact on health service utilisation. Although plans are currently underway neither the USA nor the UK have well-established guidelines which indicate appropriate contact use or receipt of health services, [50,51]. Currently, it is not clear who should co-ordinate care plans and what they should entail [52]. Overall, personalised care plans and an active role played by physicians were significant enablers in terms of helping match services to the needs of CSs and facilitating co-ordinated care – at least for particular groups of survivors.

## Abbreviations

CS: Cancer survivor; STROBE: STrengthening the Reporting of OBservational studies in Epidemiology; CEA: Carcino-Embryonic Antigen testing; ACSO: American Society of Clinical Oncology; FOBT: Faecal Occult Blood Test; PSA: Prostate Antigen Testing; CES-D: Centre of Epidemiological Studies-Depression scale.

## Competing interests

The authors do not have any financial, professional or personal conflicts to declare that are relevant to this manuscript.

## Authors’ contributions

CT was responsible for implementing the search strategy, screening papers/studies for inclusion and exclusion and drafting the manuscript under MD’s supervision. Both authors read and approved the final manuscript.

## Pre-publication history

The pre-publication history for this paper can be accessed here:

http://www.biomedcentral.com/1472-6963/12/316/prepub
